# Multidisciplinary investigation of two Egyptian child mummies curated at the University of Tartu Art Museum, Estonia (Late/Graeco-Roman Periods)

**DOI:** 10.1371/journal.pone.0227446

**Published:** 2020-01-16

**Authors:** Ester Oras, Jaanika Anderson, Mari Tõrv, Signe Vahur, Riina Rammo, Sünne Remmer, Maarja Mölder, Martin Malve, Lehti Saag, Ragnar Saage, Anu Teearu-Ojakäär, Pilleriin Peets, Kristiina Tambets, Mait Metspalu, David C. Lees, Maxwell V. L. Barclay, Martin J. R. Hall, Salima Ikram, Dario Piombino-Mascali

**Affiliations:** 1 Institute of Chemistry, Faculty of Science and Technology, University of Tartu, Tartu, Estonia; 2 Institute of History and Archaeology, Faculty of Arts and Humanities, University of Tartu, Tartu, Estonia; 3 University of Tartu Museum, Tartu, Estonia; 4 Estonian Forensic Science Institute, Tallinn, Estonia; 5 Institute of Genomics, University of Tartu, Tartu, Estonia; 6 Institute of Molecular and Cell Biology, Faculty of Science and Technology, University of Tartu, Tartu, Estonia; 7 The Natural History Museum, London, United Kingdom; 8 Department of Sociology, Egyptology and Anthropology, American University in Cairo, New Cairo, Egypt; 9 Department of Ancient Studies, Stellenbosch University, Stellenbosch, South Africa; 10 Department of Anatomy, Histology and Anthropology, Institute of Biomedical Sciences, Faculty of Medicine, Vilnius University, Vilnius, Lithuania; University of Florence, ITALY

## Abstract

Two ancient Egyptian child mummies at the University of Tartu Art Museum (Estonia) were, according to museum records, brought to Estonia by the young Baltic-German scholar Otto Friedrich von Richter, who had travelled in Egypt during the early 19th century. Although some studies of the mummies were conducted, a thorough investigation has never been made. Thus, an interdisciplinary team of experts studied the remains using the most recent analytical methods in order to provide an exhaustive analysis of the remains. The bodies were submitted for osteological and archaeothanatological study, radiological investigation, AMS radiocarbon dating, chemical and textile analyses, 3D modelling, entomological as well as aDNA investigation. Here we synthesize the results of one of the most extensive multidisciplinary analyses of ancient Egyptian child mummies, adding significantly to our knowledge of such examples of ancient funerary practices.

## Introduction

**Fig 1:**
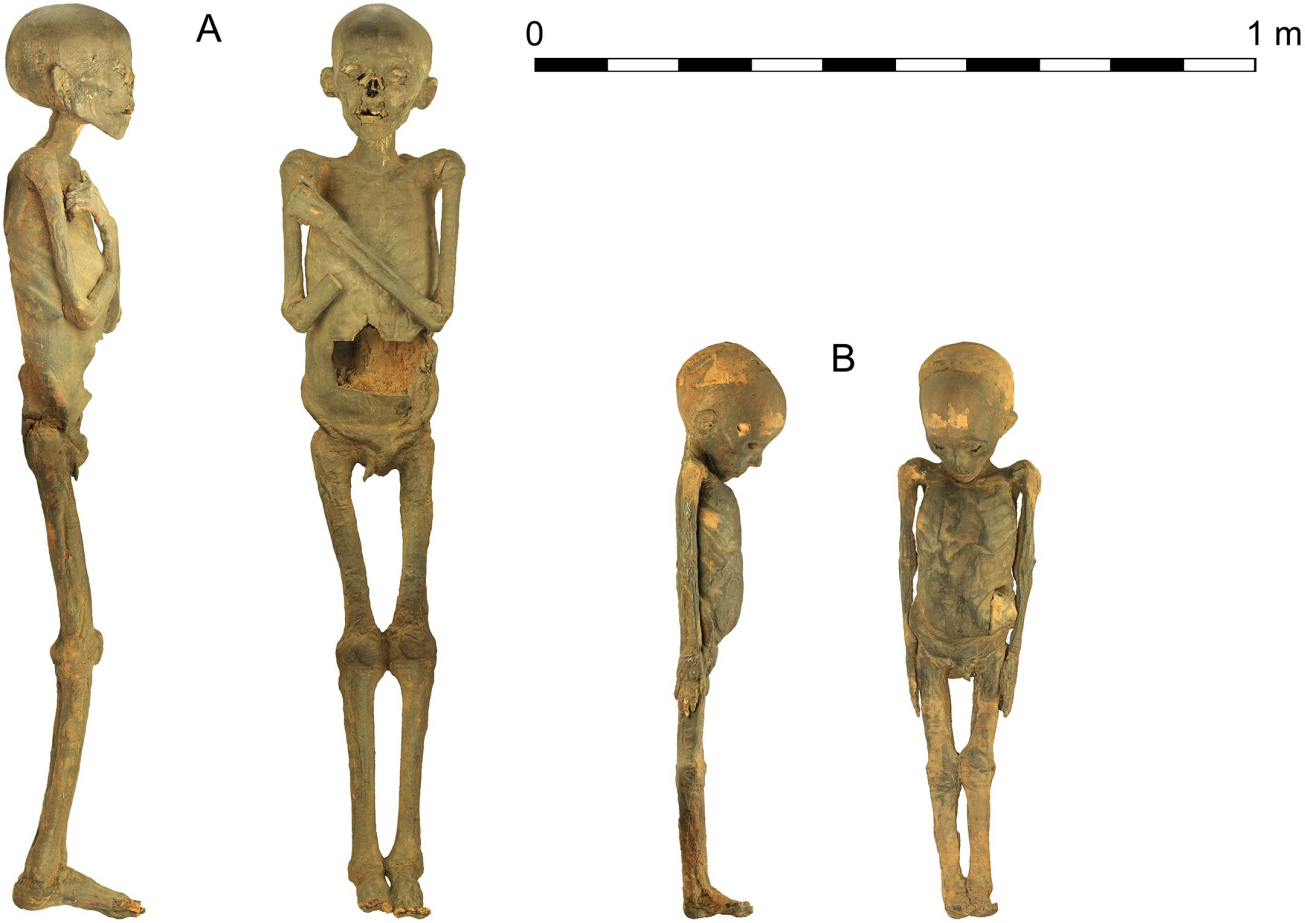
The two human mummies from the collections of the University of Tartu Art Museum. A—older mummy (OM; KMM A 64); B—younger mummy (YM; KMM A 63).

Two human mummies (Fig 1) are stored and exhibited in the University of Tartu Art Museum collections. According to museum records, Otto Friedrich von Richter, a young Baltic-German scholar and traveller, brought them to Estonia from Egypt in the early 19th century. Although these mummies were previously studied in the early 20th century, as evidenced by their appearance, many details of their origins, date, and contents are largely unknown. Thus, a team of over 20 experts from different fields such as archaeology, medicine, and the natural sciences, examined the remains using the most recent analytical methods. Following a thorough and extensive investigation of the two human mummies, the aim of this article is to exemplify the advantages and fruitfulness of combined multidisciplinary analysis for attaining the maximal information on objects in museum collections, and to introduce the results obtained from the University of Tartu mummy specimens to a wider audience.

## Historical background

Napoléon Bonaparte’s expedition to Egypt (1798–1801) and the decipherment of the Rosetta Stone revealed the country’s rich heritage to the world. In the following decades European travellers visited the ancient sites and collected antiquities. The Baltic-German elite of Estonia also participated in this activity. Otto Friedrich von Richter (1792–1816), born in the Vastse-Kuuste (German = Neu-Kusthof) manor of South-Estonia (Livonia), was a young traveller interested in ancient cultures [1]. He studied at the Universities of Heidelberg and Vienna. After diversifying his language skills, he carried out scientific expeditions to Egypt, Asia Minor, Greece, and Lower Nubia [2]. His travels began in 1814, when he went from Odessa to Constantinople (Istanbul), where he met Sven Fredrik Lidman (1786–1845), a preacher at the Swedish legation. Lidman became his travel companion, and they were among the first European investigators of historic Nubian monuments. Since the political situation in the area was complicated, they left Egypt in the summer of 1815, taking a number of antiquities and manuscripts, whose provenance is unknown, with them. Lidman returned to Constantinople directly, but von Richter continued his travels alone in 1816, visiting cities like Damascus, Palmyra, and Aleppo. During the six-week sojourn in Constantinople he sent most of his collected manuscripts and antiquities to Sweden; from there, they were later brought to Väimela (German = Waimel), his home manor in Estonia [3]. Otto von Richter’s sudden death in the summer of 1816 in Smyrna (Turkish = Izmir), Asia Minor, brought his travel and promising academic career to an end [4]. In 1819, von Richter’s collection of Egyptian antiquities, consisting of over 120 Egyptian objects including two human and two votive animal mummies, was donated to the University of Tartu (UT; German = Kaiserliche Universität zu Dorpat) by his father Otto Magnus von Richter (1755–1826), in memory of his son, the young orientalist, to encourage future generations to undertake similar scientific endeavours [5, 6]. The provenance of the human mummies is unknown, but it is quite possible that they originated from either the Memphite or Luxor areas, the sources for several mummy collections. They could also be from the Fayum, which is particularly known for having many child mummies [7–10].

Based on changes made to the policy of the museum collections, the mummies were moved to the university’s anatomical theatre in 1862 [11]. Subsequently, they were measured, autopsied, and the bodies were briefly described. According to archival sources, the older child (KMM A 64) was probably unwrapped during the first decade of the 20th century. During this time, two pieces of cloth displaying images of mummiform gods were found on the body. They most likely depict two of the Sons of Horus, who are protective deities associated with safeguarding the deceased. Another square of linen was inscribed with the wadjet eye, known more commonly as the Eye of Horus, serving as a protective amulet on the left side, close to where the embalming incision was made. Horus eye plaques or amulets were traditionally placed over the area of the embalming incision in mummies [12–14]. In addition, two bandages, inscribed in hieratic, a cursive form of the Egyptian language, were also found on the body. The texts are of Ptolemaic date (C. Geisen, A.-K. Gill, and M. Smith, personal communications, 2019) and consist of prayers for the deceased to achieve a successful afterlife. The detailed association between the inscribed textiles and images with the mummy remains unknown due to the poor documentation of these early studies. However, the texts and images would suggest a Ptolemaic or later date for the mummy.

About twenty different samples were taken from the mummy via destructive sampling: the right forearm was removed; a tooth (upper right incisor) pulled out; the torso was cut open and mummified organs wrapped within four packages were removed; part of the lower lip was also excised, and several toes had been damaged. The study mentions the presence of white crystals on the nape, and several insect cocoons near the right ear and underneath the body. Additionally, some small samples of hair, skin, resin, and salt were removed and packed into paper. Nothing was done with these samples, as far as we know. The younger child (KMM A 63) was less damaged, with some hair samples removed, and a hole made on the right side of the head. The mummies were stored at the anatomical theatre until 1980, when they were returned to the university’s Art Museum. Today, the mummies are exhibited in the ‘mummy chamber’ of the UT Art Museum. The wider interest in these cultural objects and the need to further develop the exhibition triggered the demand for a detailed analysis of these unique items.

## Materials and methodology

The older child mummy (OM, KMM A 64), measuring 127 cm in length, lies in a supine position, with the arms crossed on the chest. The body has a dark colour, and the external genitals are still recognizable. Evidence for mummification is apparent as the body has a left flank evisceration incision measuring 9.5 × 13.5 cm, foreign material was introduced into the eye sockets, and linen fragments, probably the remains of bandages, are attached to the skin. Outer damage in the form of loss of soft/hard tissue substance is noted at the level of the nose, lips and the crowns of the two left upper incisors, and is followed by the post-mortem loss of the right upper incisors and canine. Some lines of enamel hypoplasia can be distinguished at the level of the lower central incisors. The ears appear plugged with foreign material. A number of longitudinal scratches are visible on the neck, and the feet appear to be damaged and lacking some elements at the toe level. A rectangular section of tissue on the abdomen of around 7.5 × 11.5 cm, as well as the surgical removal of the right hand and part of the related forearm, are evidence of the early 20th century autopsy.

The younger child mummy (YM; KMM A 63), measuring 80 cm, also lies in a supine position, but its arms are extended along the sides of the body. The skin is also dark, possibly due to the embalming agents, and the external genitals are still preserved. Lack of soft tissue is recognizable at the forehead and right temple level. The eyes appear wide open, with the globe completely collapsed, and some hair of a reddish colour is evident on its head. Evidence of an embalming treatment is indicated by a 7 × 3 cm long left flank incision, which is enlarged at its proximal end, and is accompanied by a fragment of linen attached to the body. Residual fragments of fabric are seen on the skin of the mummy, especially at the level of the parietal/occipital area and the lower extremities. Except for its past unwrapping, the overall preservation of the body is better than KMM A 64, with only minor damage.

During the course of this study a total of 34 samples were collected from the two child mummies (Table 1). The aim was to investigate them using as few destructive methods as possible, by utilizing smaller sample sizes and cross-use of removed samples. All necessary permits were obtained for the described research, which complied with all relevant regulations.

**Table 1:** List of samples collected from the two mummies.

Sample ID	Sample description	Sample location	Weight (if applicable)	Type of analysis^a^
**Older mummy KMM A 64**				
OM S1	Tooth	Upper left canine		aDNA, AMS, SIA
OM S2	Brownish residue	Left arm	0.99 g	ORA
OM S3	Textile thread	Abdominal cavity, lower abdomen stuffing		ORA, text
OM S4	Embalming material	Right side of the inner abdominal cavity	99.5mg	ORA
OM S5	Textile thread	Abdominal cavity		Text
OM S6	Brownish residue	Left hand	1.00g	ORA
OM S7	Black residue	Left foot, between the second and third toe	1.00g	ORA
OM S8	Black residue	Left foot big toe	0.99g	ORA
OM S9	Sinew/bone fibre?	Right foot, third toe		ID
OM S10	Embalming material	On the right ear	24.5mg	ORA
OM S11	Tooth	Upper right incisive (half)		SIA
OM S12	Textile	Back of the right lower arm	79.6mg	ORA
OM S13	Textile	Abdominal cavity	210mg	AMS
OM S14	Textile	Inner side of the right upper arm	73.9mg	ORA
OM S15	Embalming material	Inner surface of the removed abdominal skin	18.8 mg	ORA
OM S16	Textile	Abdominal cavity	31.5 mg	ORA
OM S17	Piece of skin	Piece of lip (removed during previous studies)	7.2 mg	MicroB
OM S18	Dust residue	Dust from the storage/exhibition box	23.2 mg	MicroB
OM S19	Textile	Beneath the body		Text
**Younger mummy KMM A 63**				
YM S1	Textile	Lower crown		Text
YM S2	Hair	Nape	50mg	SIA
YM S3	Textile	Under the left foot		Text
YM S4	Textile	Under the left foot		Text
YM S5	Hair	Nape	101.6mg	AMS
YM S6	Textile	Lower crown		Text
YM S7	Hair	Nape	149mg	aDNA
YM S8	Textile	Left side of the crown		Text
YM S9	Black residue from hair	Hair, crown	1.00g	ORA
YM S10	Nail	Fourth finger of the left arm	11.8mg	ORA, SIA
YM S11	Textile	Left ankle	935mg	AMS
YM S12	Textile	Textile from abdomen stuffing		Text
YM S13	Black residue	Left upper arm	1.00g	ORA
YM S14	Embalming residue	Left temple	41.7mg	ORA
YM S15	Soaked textile	Under right foot	86.5mg	ORA
YM S16	Hair	Nape	1.00g	aDNA
**Textile collection**				
T S1	Textile	Textiles		Text
T S2	Textile	Textiles		Text
T S3	Textile	Textiles		Text
T S4	Textile	Textiles		Text
T S5	Cocoons (2), fragments of beetles	Exhibition coffin of the older mummy, between and underneath textile fragments		Ent

^a^aDNA—ancient DNA analysis, AMS—radiocarbon AMS dating, Ent—entomological analysis, ID—general identification, MicroB—microbiological analysis, ORA—organic residue analysis, SIA—stable isotope analysis, Text—textile analysis (microscopic, ATR-FT-IR as applicable).

AMS radiocarbon dating was carried out at the 14Chrono Centre of Queen’s University, Belfast, United Kingdom, with carbon and nitrogen stable isotope analysis of applicable material also provided. The AMS dates were calibrated with the OxCal 4.3.2 [15], using the IntCal13 calibration curve [16] and rounded by ten.

Computed tomography (CT) scans and radiographic images were taken to reconstruct the biological profiles, estimate the age at death, and note any skeletal changes caused by disease or injury [17]. Images were created via radiography and a 3D model at the Department of Archaeology, UT. Multislice CT scans (Siemens SOMATOM Emotion 6) were performed at the Estonian Forensic Science Institute (EFSI). Initial axial CT scans were obtained at a tube voltage of 130 kV with automatic tube current modulation and with slice thickness of 0.63–0.75 mm. Anteroposterior and lateral topograms were obtained first. The older child (KMM A 64) was scanned inside the coffin in which he is exhibited. A scan from the head to the upper thighs and a separate scan from the pelvis to the feet were obtained, followed by a scan of the head and neck. The younger child (KMM A 63) was scanned without a coffin, first from the head to the calves, followed by a scan from the head to the feet. Multiplanar reformatted images and 3D reconstructions were subsequently created. Reading and post-processing was carried out using syngo.via software (syngoMMWP VE40A, Siemens AG). Density of possible foreign materials was described based on the Hounsfield Unit scale (HU) [18].

Despite the soft tissues having dried significantly during mummification, it was possible to determine the sex based on the inspection of external genitalia. The age at death was estimated based on dental development, development of the long bones, and epiphyseal fusion [19, 20]. For the archaeothanatological study, based on the precepts laid out by Duday [21], CT scan images identifying bone lateralization and position, combined with visual observations and taphonomic aspects, were taken into account to reconstruct the initial burial practices and the treatment of the body during and after the mummification process. Although archaeothanatology is essentially a field method for primary excavation [21], it can also be applied to old excavation data in order to obtain more information from them [22–26].

Ancient DNA (aDNA) extraction was performed in a dedicated aDNA laboratory at the UT, Institute of Ecology and Earth Sciences, Estonia (see S1 Appendix A for details), and sequenced with the Next-generation Sequencing technology on Illumina platform. Sequencing libraries were built using NEBNext DNA Library Prep Master Mix Set for 454 (E6070, New England Biolabs) and Illumina-specific adaptors, following specific protocols [27]. The libraries were shotgun-sequenced at the UT Institute of Genomics, Estonian Biocentre core lab with Illumina NextSeq 500 using a 75 bp single-end kit. The program mapDamage2.0 was used to ensure that DNA damage patterns were characteristic of aDNA [28].

Organic and inorganic residue analysis of various embalming materials was carried out at the Institute of Chemistry, UT, using optical microscopy, ATR-FT-IR spectroscopy (attenuated total reflectance—Fourier transform—infrared spectroscopy), GC-MS (gas chromatography—mass spectrometry), ESI-FT-ICR-MS (electrospray ionization—Fourier transform—ion cyclotron resonance—mass spectrometry), and SEM-EDS (scanning electron microscopy—energy dispersive X-ray spectroscopy). A more detailed description of the used instruments and experimental information can be found in the Supplementary Material (S2 Appendix B).

The textile analysis was conducted at the Department of Archaeology, UT, using transmitted and polarised light microscopy, as well as SEM (scanning electron microscopy). The textile fibre studies were conducted using different microscopy techniques. In addition to the optical transmitted light microscope, polarised light was employed (Olympus BX-51P up to 500× magnification) and the determination of the fibrillar orientation of bast fibres was conducted using the modified Herzog test [29]. Occasionally, scanning electron microscopy (SEM; Zeiss Sigma VP at Aalto University Nanomicroscopy Centre, Finland) was also employed. The identification was based on a comparison with reference collections and on the works of Catling and Grayson [30] and Rast-Eicher [31]. Besides traditional light microscopy, some of the textile samples were analysed at the Institute of Chemistry using ATR-FT-IR spectroscopy and classification based on principal component analysis (PCA) [32].

The archaeo-entomological study was conducted at the Natural History Museum, London [33]. The cocoons were photographed from both surfaces using a Canon^®^ MP-E 65mm f/2.8 1–5 Digital Picturelens at 5× and using a motorised drive for stacking of around 50 individual images in Helicon Professional^®^ software. They were then cut open with micro-scissors along one side to check for contents. The head capsule was measured using a Leica^®^ M165 stereomicroscope equipped with Leica^®^ DFC295 camera combined with Leica Software Application Suite (LAS). In addition, fragments from beetles (cast larval skin) were visually examined.

Finally, both mummies were 3D-modelled (see https://skfb.ly/6u8Hu, https://skfb.ly/6HrZO for the older and younger mummy, respectively) using photogrammetry (see S3 Appendix C for analytical details) in order to document their present state and to create an interactive 3D model for the museum. The models were photographed with a Canon EOS 600D SLR camera operating with a standard 18–58 mm lens. Two different photographic approaches were tested for comparison. The younger mummy (KMM A 63) was supported on the edge of two Plexiglas panels from the neck and knee area, and then photographed for a single model. The older mummy (KMM A 64) was photographed for two models: lying on its back and then on its stomach, which would later be combined. The models were made using Agisoft PhotoScan Pro. Ed. (v. 1.3.4).

## Results and discussion

### Dating

**Fig 2:**
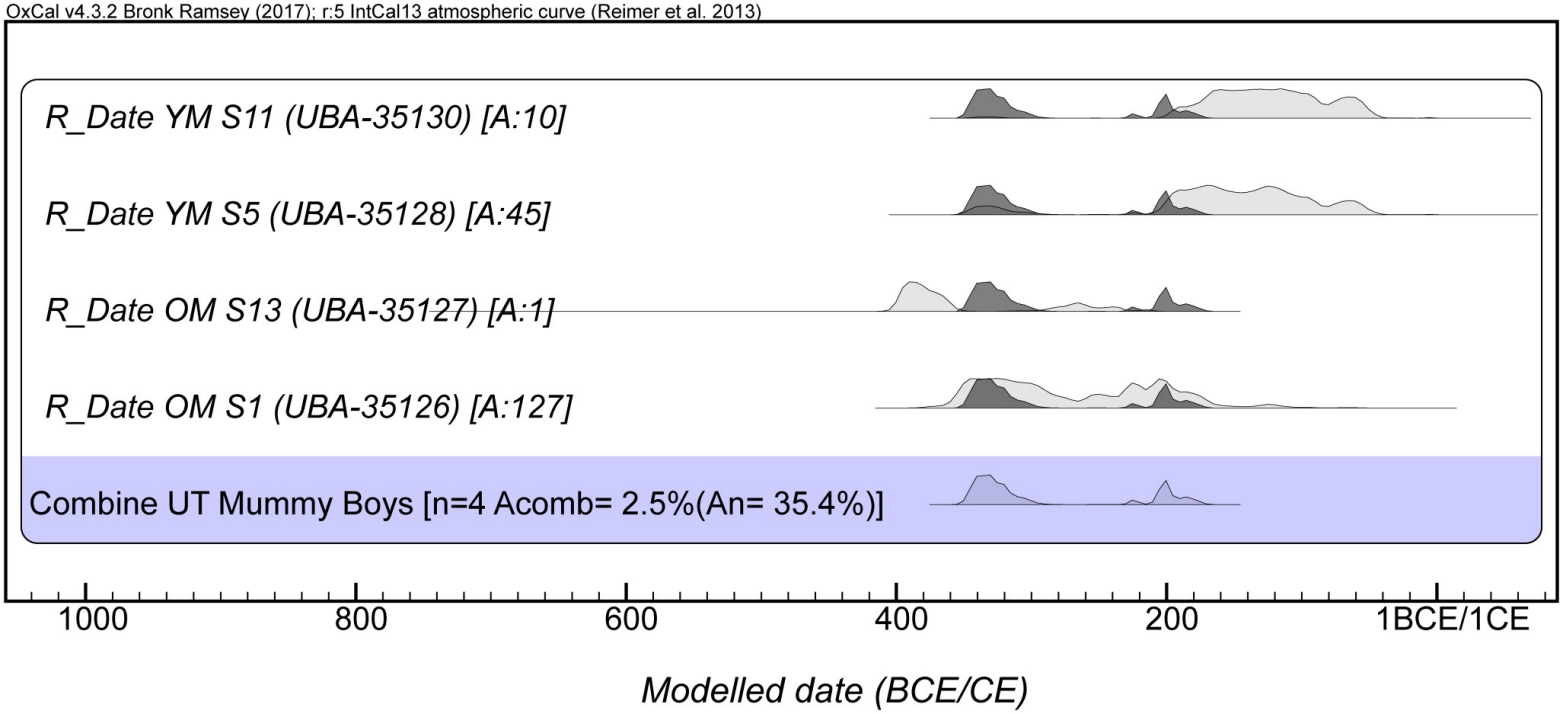
The calibrated dates of the two mummies and the OxCal4.3.2 combine model shows that the subjects were not buried at the same time.

Four samples (Table 2; Fig 2) were AMS-dated to establish when the mummies were prepared. Textile wrappings, human hair, and tooth samples were comparatively analysed. The calibrated dates of the two subjects placed them within the second half of the first millennium BC: specifically, the older subject (KMM A 64) dates to the end of fifth until the first half of the second century BC, while the younger one (KMM A 63) dates to the mid-fourth until the mid-first century BC. These dates agree with the dating of the texts found on the bandages. It is also noteworthy that the textile and human tissue results agree, suggesting that coeval textiles were used for wrapping the bodies. The calibration model shows some overlap of the dates (mid-fourth to mid-second century BC), raising the question of whether the mummies might have been buried around the same time. To test the hypothesis of a single event, the application of Combine function in the OxCal 4.3.2 program was used. The poor indices of agreement (n = 4; A_comb_ = 2.5%; A_n_ = 35.4%) of the model do not validate this assumption. Thus, it is clear that these bodies were not deposited simultaneously, although both were embalmed and buried between the end of the fifth to the second half of the first century BC, with the older child (KMM A 64) being buried slightly before the younger one (KMM A 63).

**Table 2:** AMS dates from the mummies. The AMS dates were calibrated with the OxCal 4.3.2 [15], using the IntCal13 calibration curve [16] and rounded by ten.

Sample ID	Dated material	^14^C Age	Calibrated Age (2δ)	Lab no.
**Older mummy** (KMM A 64)				
OM S1	tooth (root)	2181 ± 32	370–160 BC	UBA-35126
OM S13	textile	2294 ± 31	410–230 BC	UBA-35127
**Younger mummy** (KMM A 63)				
YM S5	hair	2122 ± 32	350–40 BC	UBA-35128
YM S11	textile	2103 ± 26	200–50 BC	UBA-35130

### Imaging

The skin, subcutaneous soft tissues, and muscles were desiccated in both mummies; the surviving outer genitalia identified both children as males. The age at death of KMM A 64 was estimated to be between 11 and 15 years old, and KMM A 63 was estimated to be between 2 to 4 years old.

**Fig 3:**
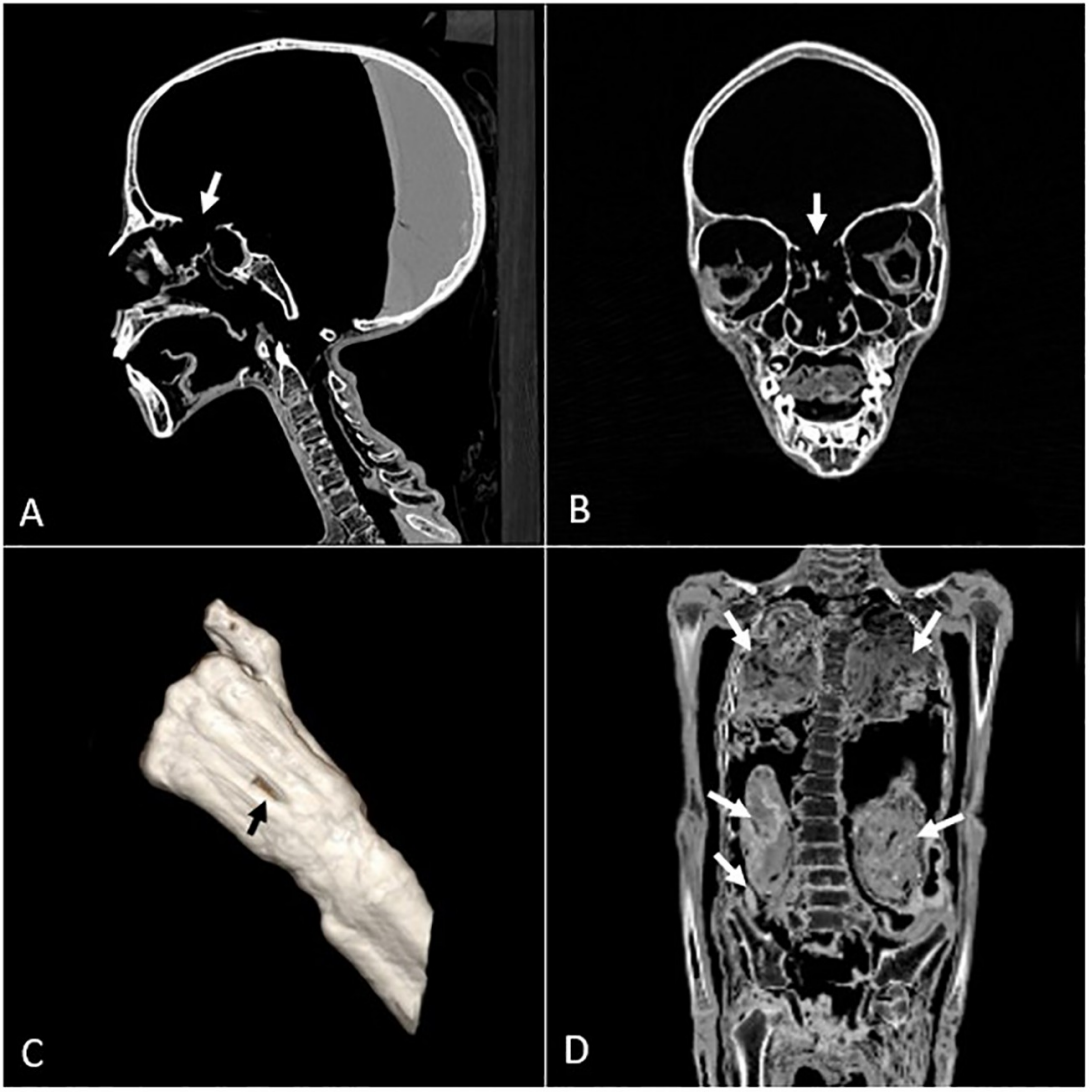
CT scan images of the mummies. A—trans-nasal craniotomy in the form of an anterior skull base defect and solidified resin inside the skull of the older mummy; B—anterior skull base defect in the younger mummy; C—linear skin defect on the back of the left hand of the older mummy; D—torso of the younger mummy showing folded textiles inside the thoracic cavity, as well as textile bundles and a small oval object inside the abdomen.

Transnasal craniotomy had been performed on KMM A 64, resulting in a broken nasal septum with bony defects in the anterior skull base (cribriform plate) [34]. No clearly identifiable brain tissue was noted inside the cranium; instead, inhomogeneous solidified resinous material filled approximately one third of the cranial cavity (Fig 3A). This did not extend into the cervical canal. The resinous material had a layered appearance, with the dense uppermost layer measuring 160 HU in average, the intermediate layer measuring 60 HU in average, and with high-density (up to 300 HU) debris at the bottom [35, 36]. Several voids were apparent inside the resin, mostly on the left side, with the largest measuring up to 6 × 2 cm in the axial plane and 11 cm in the craniocaudal plane; these could possibly be caused by the remnants of the dural membranes. The head of the mummy was slightly turned to the right, which was consistent with the levels emerging between the layers of different density resin. However, the upper air-resin level inside the skull was diagonal with the left side being significantly higher, and some of the voids presented a sagittal air-resin level. Thus, it would seem that resin was poured into the skull more than once, with the angle of the head being slightly changed between the two events.

A small amount of resin could also be seen inside the paranasal sinuses of KMM A 64. Inhomogeneous layered tissue with average densities of 300–200 HU was noted inside the orbits [35]. It is not clear whether these represent orbital contents or, most likely, foreign material. The external ear canals were partially obliterated, possibly by textile plugs. Parts of the lips appeared to be absent, as well as both of the right upper incisors. Both of the left upper incisors were broken. Both maxillary and mandibular third molars had not erupted. A well-defined cystic lesion with cortical thinning but no absorption of roots or mass effect on the adjacent teeth could be seen on the right side of the mandible in association with the roots of the right lower incisors, canine and premolars, measuring approximately 1.9 × 0.9 cm on the axial plane [37]. A structure resembling a desiccated tongue was attached to the floor of the mouth. A disruption of the ligaments between the skull base and upper cervical vertebrae was visible, as well as a defect in the pharyngeal and prevertebral tissues. These resulted in an atlanto-occipital dislocation with an abnormally wide basion-dens interval (1.3 cm) and created a connection between the pharynx and spinal canal/cranial cavity. Remnants of the dural membranes could be seen inside the spinal canal.

The thorax and abdomen were slightly deformed with the left side of the body compressed. Irregular defects could be seen in the abdominal wall above the incision as well as to the left of the incision, with the one on the left being related to the original evisceration [12]. Remnants of the mediastinum could be identified with some tissue, possibly a lung fragment, attached to the left side. Linear opacities, likely the diaphragm remnants, were also identified. The rest of the thoracic and abdominal organs were absent, most likely due to evisceration. Conventionally, the heart was left in the body, but this mummy showed no evidence of this practice. It might have been removed accidentally, or was desiccated and firmly embedded in the resinous substance, thus rendering it invisible. Folded textile or a fragmented organ package was seen inside the left side of the pelvis, but no internal organs could be positively identified within them. Originally the mummy contained four packages, possibly one for each organ that was traditionally removed during the course of mummification (lungs, liver, stomach, intestines). These were often mummified separately, and then at various periods in Egyptian history, would have been returned to the body.

A layer of resin with an average density of 130 HU could be seen along the dependent part of the thoracic and abdominal wall. A thicker layer of resin could be seen on the right side of the body cavity and, similar to the findings inside the skull, the level of the resinous material was diagonal, suggesting that the body had been tilted towards the right side while the resin solidified. A layer of resin of similar density was also identified inside the spinal canal, possibly related to the resin along the thoracic and abdominal wall, with the intervertebral foramina being the likely connection. Heterogeneous, mostly unidentifiable debris was noted on top of, as well as within, the resin. This included thin objects with an average density of approximately 1500 HU, some of which had a triangular shape and were likely pieces of glass that were the result of storage issues. Similar objects could be seen inside the textiles lining the coffin, and might have been introduced accidentally during the studies on the mummy carried out in the early 20th century. There appeared to be a linear defect on the skin of the back of the left hand, in the area between the III and IV metacarpal bone measuring approximately 1 cm, which was taphonomic in origin (Fig 3C).

The pelvic bones were disarticulated and dislocated. The feet were not intact. On the right foot only the I proximal phalanx with the epiphyseal plate of the distal phalanx was present, as well as the epiphyseal plate of the proximal phalanx of the II toe, a part of the proximal phalanx of the IV toe, and a complete proximal phalanx of the V toe. On the left foot all of the proximal phalanges were present, the III toe also had a partial middle phalanx, the IV toe had a complete middle phalanx, and the V toe had all three phalanges. The missing phalanges could have been lost during the alleged 20th century autopsy of the mummy.

The younger child (KMM A 63) was prepared in a way that was similar to the older one (KMM A 64). The brain had been removed with a transethmoidal approach resulting in a bony defect in the nasal septum and the anterior skull base (Fig 3B) [34]. In KMM A 63 fragments of a substance thought to be solidified resin (~ 450 HU) were seen inside the skull and the spinal canal. There was layered tissue inside the orbits and the oral cavity, the latter probably being packing material. No remnant of the tongue could be identified. A post-mortem soft tissue defect in the scalp of the right temporal region was clearly visible. The maxilla and mandible both showed 10 deciduous teeth, with the exception of the right mandibular lateral incisor; the missing tooth was seen on the floor of the mouth on the right side. The right side of the maxilla showed five unerupted permanent teeth. The left side of the maxilla showed six unerupted teeth, and the supernumerary tooth was in the midline above the left deciduous central incisor. The mandible showed 10 unerupted permanent teeth, all of which contributed to the estimate of the child’s age. The cervical spine had a kyphosis and there was a gap between the II and III thoracic vertebra. On the coronal plane, there was a slight thoracolumbar spinal curvature to the right, about 13°. The coccygeal vertebrae were not visible. There was fragmented resin inside the spinal canal, mostly on the posterior side and on the sides of the canal; some residual tissue could also be seen. The ribs were separate from the costal cartilage and most of the ribs and cartilages were dislocated. Evisceration had been performed through an incision on the left side of the abdomen, as is usual. None of the internal organs could be identified loose in the body cavity. As was the case with KMM A 64, the heart could not be identified in the CTs. Unlike the older child, the thorax was tightly packed with layered material, probably folded and wadded textile, and some of the material had a striped or corded texture on oblique reconstructions (Fig 3D). Two packages of rolled textile possibly containing embalmed organs had been placed inside the abdomen—the older child had four such packages originally. In addition to the packages, there was a smooth oval object with a groove on one side, with a diameter of 0.7 cm in the axial plane, a length of 2.5 cm, and a density of approximately 350 HU. This might have been an amulet of some kind, or maybe a bead. Fragmented resin (~ 350–400 HU) was visible inside the abdomen and pelvis, more on the left side, where it partly enclosed one of the packages. Layers of textile, similar to that in the thoracic cavity, could be seen inside the pelvis. The opening on the left side of the abdominal wall was partially closed by the textiles and resin. The pelvic bones were disarticulated and dislocated. The extremities seemed to be intact, although, the toes were difficult to examine due to remnants of textiles. CT images also revealed a single line in the distal third of both femoral bones, located at the same level. They were identified as Harris lines, usually considered to be the result of nonspecific stress such as disease or malnourishment [38]. Since the bones continued to grow, the biological stress that the child experienced was not fatal.

### Archaeothanatology

Mummification causes the soft tissue to resist decay through desiccation of the corpse [39]. This means that the bones maintain their anatomical position, indicating that the body position they are discovered in largely reflects their final position during the embalming process. However, the archaeothanatological observations revealed several hidden aspects regarding the positioning of these bodies.

The older mummy (KMM A 64) was lying on its back, head looking straight forward, his upper limbs were tightly adducted at the shoulders, flexed at the elbow, with the arms crossed over the chest with the right one placed over the left. The wrist of the left hand was extended, but the fingers were flexed at the second and third interphalangeal joint. As the right hand was missing (see above), its position is unknown. The position of the left hand and fingers being flexed at the second and third interphalangeal joints and the distance between the fingers and palm suggest that the body originally might have held something. A left clenched hand has been reported in a number of mummies, and appears to be common during the Late Period [12, 40], but the reasons behind this practice have not been confidently ascertained. It has been posited, however, that they were holding an object, as has been evidenced by some mummies [41]. The legs were extended and placed close together, probably as they were tightly wrapped; the feet were lying parallel. The arching lower back and lower limbs suggest that some sort of supportive element was placed behind the lower back to the distal end of the legs, most likely after the body was wrapped in layers of linen and placed inside a coffin or buried. Whether the supportive element was the outcome of a cultural act (e.g. placement of a pillow-like object) or a taphonomic effect caused by the natural conditions of the burial (e.g. subsequent deposition on stones) cannot be ascertained. However, this arching of the lower back has been noted in other child and adult mummies in excavations in Thebes, where the rock-fall coupled with flooding has been posited as the cause for the positioning (S. Ikram and J. Herrerin, personal observations, 2019). The CT scan showed that his pelvic bones were disarticulated and moved towards the inferior part of the pelvic region. This suggests that at some point the corpse was placed in an upright position—there is evidence for adult mummies of the Graeco-Roman Period to be placed this way in domestic spaces for some time prior to burial [12]. Although mummification of tissues may be partial, leading to bone dislocation [26], the exact timing of these post-depositional changes at the bone level cannot be determined. The position of both clavicles (perpendicular to the midline of the body) and arms (adducted at the shoulder and flexed at the elbow, leaving the forearms in front of the torso with some space in between) suggests that the upper body was not tightly wrapped, while the position of the lower limbs is indicative of tight wrapping. In the head region, the fabric was applied in a way to force the head to face anteriorly (Fig 1A).

The younger child (KMM A 63) was lying on its back with the upper limbs tightly extended along the sides of the body. Unlike the older mummy (KMM A 64) his chin rested on the chest, a common pose in younger child mummies, particularly of the Graeco-Roman era [42–44]. The position helps keep the head secure in younger children whose vertebrae are less sturdy. Maintaining the intactness of the body, especially the head, was a significant concern of the embalmers. The child’s legs were extended, and his feet were in a neutral position or slightly dorsiflexed. The loss of the anatomical arching of the lower back and the overall position of the posterior body visible on the 3D model suggest that the subject was initially placed on an even bottom and hard surface. This fact together with the disarticulated pelvic bones that have moved posteriorly in the pelvic region during desiccation vividly demonstrate that the child was initially placed on his back and was not moved much during the time when the soft tissue and ligaments were still flexible. The overall body position—chin on the chest, upper limbs tightly next to the lateral sides of the body, and lower limbs tightly together from the hips to the heels—suggests that he was initially wrapped as a single unit, i.e. the head and body together, leaving his chin on the chest. The position of both clavicles (perpendicular to the midline of the body) and arms (adducted at the shoulder and extended at the elbow, next to the lateral sides of the body) hints that the upper body was wrapped slightly more loosely than the area inferior to the pelvis (lower limbs tightly next to one another from the hips to the heels).

### Ancient DNA

**Fig 4:**
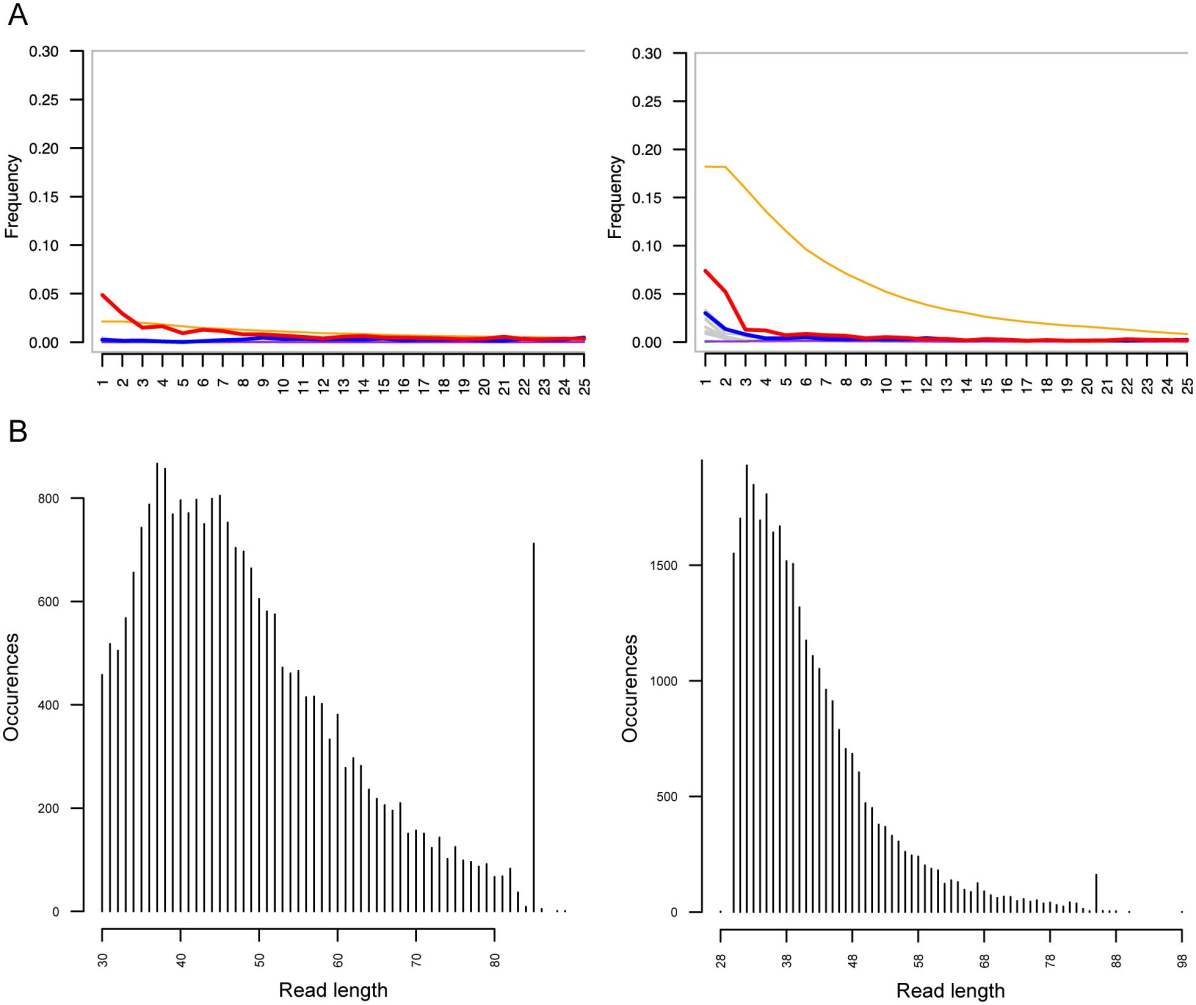
Ancient DNA authentication. Older mummy (OM S1, tooth root) on the left, younger mummy (YM S5, hair) on the right. A. DNA damage at the ends of fragments. Red line—C=>T substitutions; blue line—G=>A substitutions; orange line—soft-clipped bases. B. Read length distribution of sequencing reads mapped to the human reference sequence.

Due to poor conditions of biomolecule preservation, aDNA analysis of mummified individuals from regions with a hot and humid climate has not been very successful until recently, when a comprehensive study of the mitochondrial DNA (mtDNA) variation of 90 individuals and genome-wide data of three Egyptian mummies from the New Kingdom to the Roman Period was published [45]. The date of our mummies (Table 2) falls into the same time frame, providing a relevant context to our data (see S1 Appendix A for details). We were able to extract and sequence low-coverage (0.0004x) human genomic data with substitution patterns and read lengths characteristic of aDNA from the tooth root (OM S1) and hair (YM S7) of both mummies (Table 3, Fig 4). The proportions of endogenous human DNA in the extracts were 0.17% (OM S1) and 2.41% (YM S7), which allowed us to determine the maternally inherited mitochondrial DNA (mtDNA) haplogroups (hgs) of both individuals.

**Table 3:** Results of aDNA analysis from the two mummies.

Sample type	ID	Human DNA	Clonality	Effectivity	Human reads (MQ>10)	Average coverage	Average read length	5' C=>T	λ	δs	mtDNA hg
tooth	OM-S1	0.17%	32.43%	0.06%	23603	0,0004x	50 bp	4.87%	50.36%	17.64%	T2c1a
hair	YM-S7	2.41%	58.27%	0.08%	31455	0,0005x	45 bp	7.38%	56.01%	38.86%	HV

The mtDNAs retrieved from the mummies belong to hgs T2c1a (OM S1) and HV (YM S7). Identical or phylogenetically close derivatives of these lineages are present in both ancient and modern Egyptians as well as among several present-day populations of the Near East and North Africa [45–51]. Haplogroup T2c has a Near Eastern origin and T2c1 is the most frequent clade of T2c [49]. The highest frequencies (6%) of T2c1 have been found in Cyprus but it is relatively frequent (1–2%) also in the Levant and in Southern Europe (the Mediterranean coast). In the rest of Europe these maternal lineages are usually found only at very low frequencies [49]. Hg HV is spread today mostly in North Africa and West Eurasia [46–48, 51]. Notably, the highest frequency (14.3%) of hg HV has been reported among Egyptians from El-Hayez oasis but this hg is also frequent (5–8%) in the rest of North Africa [52, 53]. Although mtDNA hg alone is not enough to reach any precise conclusion about the origin of an individual, our results are in accordance with an Egyptian origin.

### Embalming materials

Embalming residues from different body areas, as well as impregnated textiles used for wrapping the bodies and stuffing the body cavities, were analysed first using ATR-FT-IR spectroscopy to identify organic and inorganic compounds. Then, GC-MS and ESI-FT-ICR-MS analyses were conducted for further organic compound and SEM-EDS for inorganic compound specification (see further details on sample preparation, instrument settings in S2 Appendix B).

**Fig 5:**
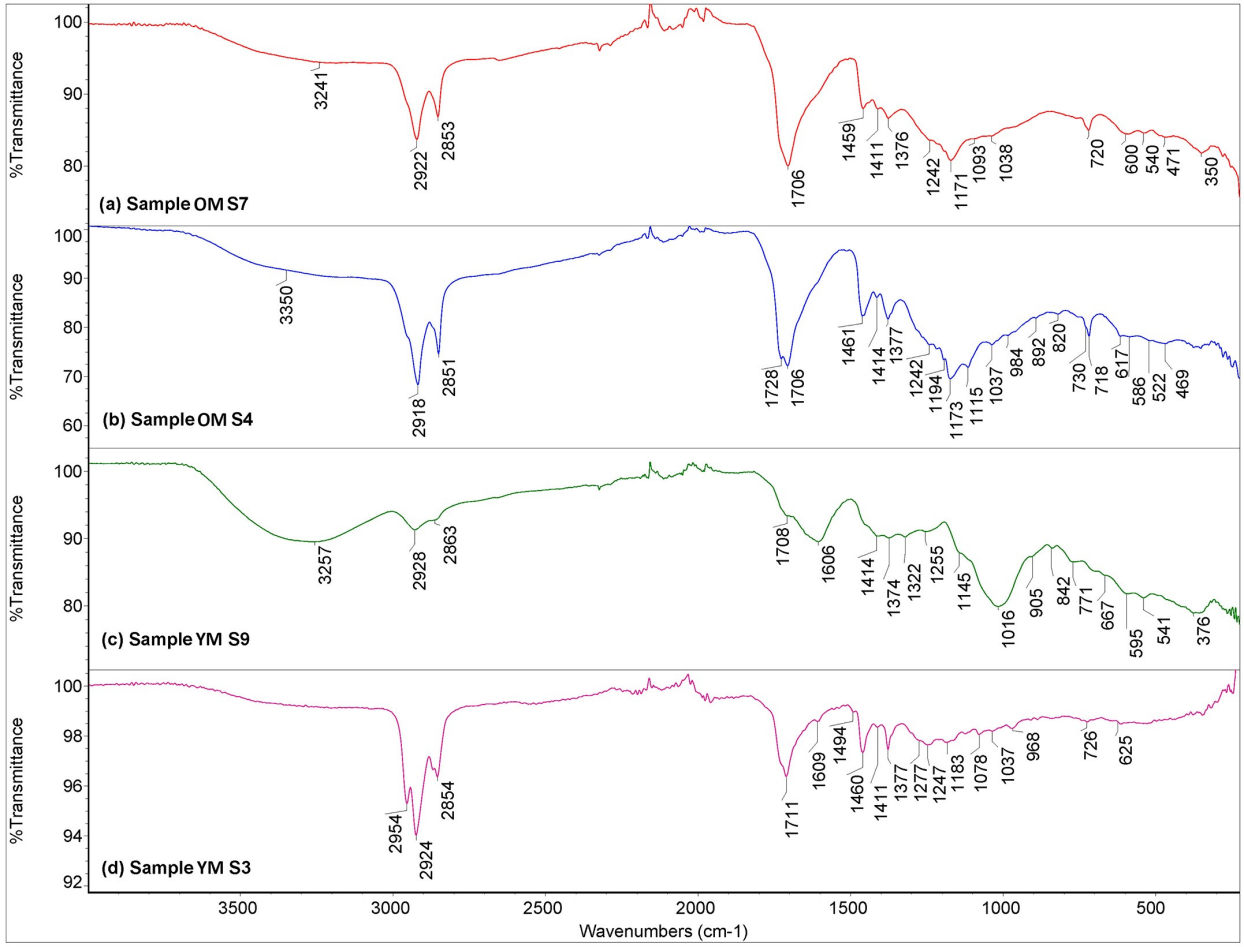
Selection of ATR-FT-IR spectra of embalming and textile impregnating materials. A—Material from the older mummy’s left foot (OM S7); B—Material from the older mummy’s abdominal cavity (OM S4); C—Material from the younger mummy’s hair (YM S9); D—Textile-impregnating material from the younger mummy (YM S3).

ATR-FT-IR analysis (Fig 5), further supported by GC-MS (see S2 Appendix B, Table 1) and ESI-FT-ICR-MS, indicates that the embalming material consists of some plant oil and/or (inclusion of) animal fat. Additionally, traces of terpenoid resins, aromatic compounds, polysaccharides, and in some samples carotenoids were also detected (see Table 4 for further specifications). In most cases we could not differentiate with certainty whether plant oil and/or animal fat had been used as the main component, because differentiation between animal fat and plant oils in old, composite and degraded materials is complicated [54]. However, some instances (e.g. sample OM S10) might be plant oils, due to the clear dominance of palmitic acid (C_16:0_) over stearic acid (C_18:0_), based on GC-MS results [55]. Inclusion of cholesterol and its derivatives that were detected with GC-MS might be part of the initial embalming mixtures, but could also derive from the human tissue itself.

**Table 4:** Materials used for the embalming of the mummies^a^.

Material	Older mummy (OM) KMM A 64	Younger mummy (YM) KMM A 63
**Embalming substances**	***On the body***: plant oil and/or animal fat resinous substance (mainly diterpenoids, low intensity of triterpenoids) polysaccharides (natural plant gum) aromatic compounds (e.g. phenolic acids: benzoic acid, cinnamic acid) coumarin carotenoids (traces) compounds connected to asphaltene, biochar proteinaceous material (traces) inorganic compounds (Na_2_SO_4_, CaCO_3_, Fe-containing compounds and silicates) ***Inside the abdominal cavity***: plant oil and/or animal fat resinous substances (diterpenoid (Pinaceae resins?), triterpenoid (mastic) beeswax aromatic compounds (e.g. phenolic acids: benzoic acid) cholesterol inorganic compounds (Na_2_SO_4_, NaClO_2_, slight traces of silicates and CaCO_3_)	***On the body***: plant oil and/or animal fat resinous substance (Pinaceae resin?) ricinoleic acid aromatic compounds (e.g. phenolic acids: benzoic acid) proteinaceous material cholesterol inorganic compounds (silicates) ***Black residue from hair***: carbohydrates (gum arabic, fruit tree extract?) inorganic compounds (silicates, CaCO_3_, some sulphates) ***On the fingernail***: plant oil and/or animal fat (?) resinous substance (Pinaceae resin?) proteinaceous material inorganic compounds (Fe-containing silicates (some ochre?), sulphates, carbonates, phosphates, Cl-containing compounds)
**Textile impregnation materials**	***On the body***: plant oil and/or animal fat wax resinous substances (?) inorganic compounds (CaCO_3_, silicates and some sulphates) ***Inside the abdominal cavity***: plant oil and/or animal fat mineral oil resinous substances (?) inorganic compounds (carbonates, silicates)	***On the body***: plant oil and/or animal fat resinous substance (Pinaceae resin?) aromatic compounds (e.g. phenolic acids: benzoic acid) mineral oil (bitumen?) polysaccharides (pyranone, furanone (arabinofuranose) and derivatives cholesterol

^a^**ATR-FT-IR**: OM S2, OM S3, OM S4, OM S6, OM S7, OM S8, OM S10, OM S12, YM S1, YM S3, YM S4, YM S9, YM S10, YM S13, YM S14; **GC-MS**: OM S4, OM S10, YM S14, YM S15; **ESI-FT-ICR-MS**: OM S4, OM S10, YM S14, YM S4; **SEM-EDS**: OM S4, OM S7, YM S9, YM S10.

**Fig 6:**
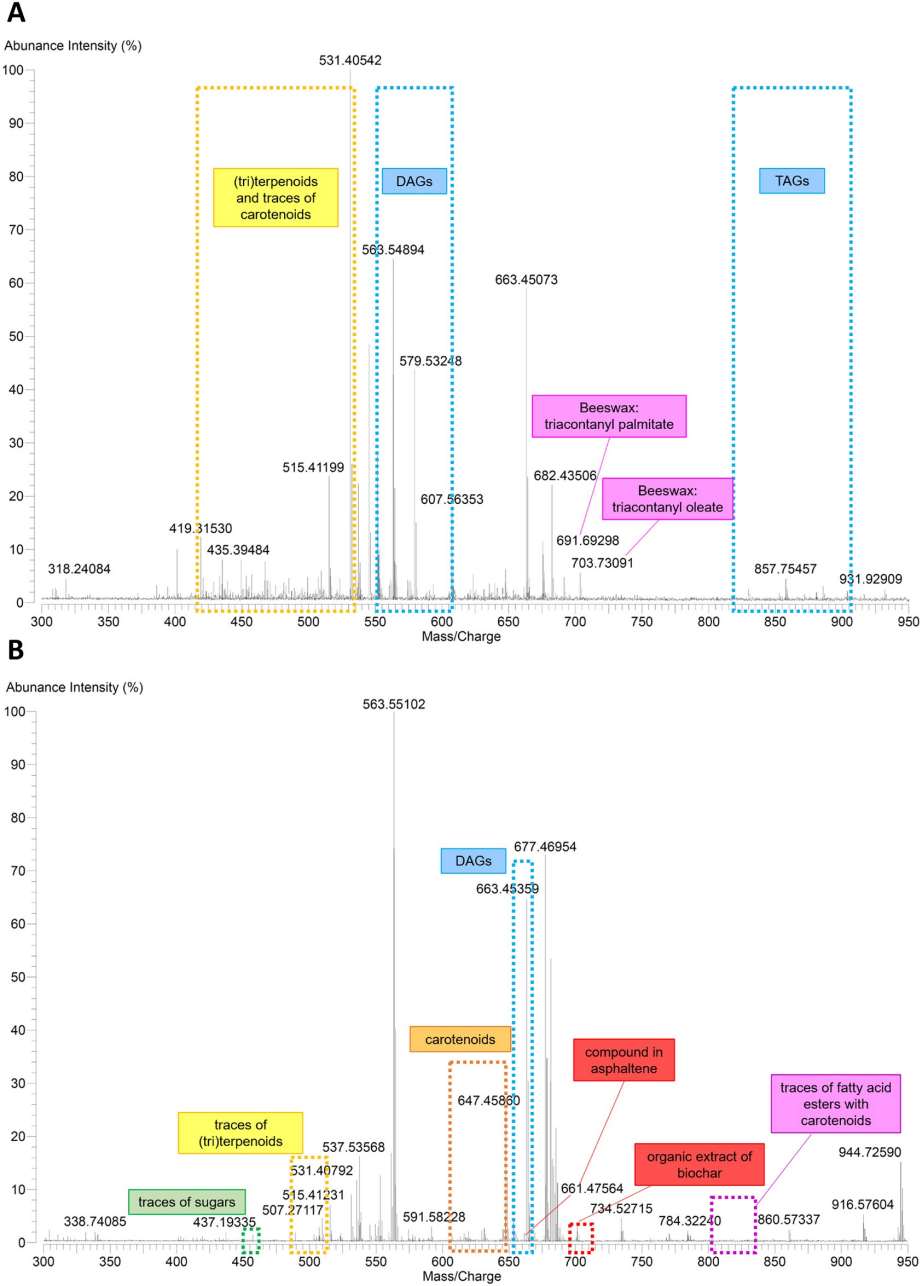
ESI-FT-ICR-MS spectra with identified compounds. A—sample OM S4; B—sample YM S4.

Mass-spectrometry provided further specification of resin components. Besides general fatty acid profiles, GC-MS analysis showed the presence of abietic acid and its derivatives, indicating conifer resins of the Pinaceae group such as cedar and pine [56, 57]. The triterpenoid resin (e.g. mastic) could not be excluded based on the numerous peaks detected within the ESI-FT-ICR-MS spectra corresponding to terpenoid compounds with 29 and 30 carbon atom skeletons (e.g. sample OM S4). Embalming material from the abdominal cavity of the older child (sample OM S4; Fig 5B) resembled the other samples by and large, but also had indicators of waxy substances based on ATR-FT-IR analysis (possibly beeswax) [58, 59]. The latter was supported by ESI-FT-ICR-MS analysis detecting triacontanyl palmitate, one of the major components in beeswax (Fig 6A). Potential of natural wax inclusion (possibly degraded beeswax) was also partially visible in GC-MS results shown by some long-chain alkanes like C_27_, C_29_ and C_44_, yet lacking n-alkanols and longer chain (>24) fatty acids [57, 60–62]. In sample OM S10 a selection of polysaccharides like pyranone, furanone and their derivatives were identified with GC-MS analysis. These could be potentially related to natural plant gum or gum-resin [60]. Coumarin, the traces of which were found in the same sample, could be related to galbanum [63]. Additionally, the ESI-FT-ICR-MS spectrum obtained from sample YM S4 displayed peaks corresponding to compounds found in asphaltene and biochar (Fig 6B), perhaps evidence for the use of bituminous material and pine tar (biochar is a byproduct of pine tar) in the embalming mixture, at least in the head/ear region of the older child.

Optical microscopy and ATR-FT-IR analysis of the younger mummy showed that his hair and body were treated with different materials. A sample taken on the left upper arm (sample YM S13, analysed with ATR-FT-IR only) probably indicates some plant oil, with possible addition of resin and silicates. Samples from the head area but also from under the nail showed substances of plant oil and/or animal fat origin, also detected with GC-MS. Cholesterol was identified with GC-MS, but this might again derive from the body tissues. Besides some aromatic compounds, traces of ricinoleic acid, possibly indicating the use of castor oil [55, 64] were found in one of the samples (YM S14). The inclusion of phenanthrene (its derivatives) together with derivatives of abietic acid showed that possible pitch from Pinaceae wood was used in the embalming [65]. The ATR-FT-IR analysis of the samples from the head area indicated that the hair was treated with some carbohydrate-containing (i.e. saccharides) materials (Fig 5C). Based on these IR spectra alone, it is difficult to further identify the exact carbohydrates represented, but they could represent some fruit tree (for example plum, cherry, etc.) or acacia tree (for example gum arabic) extracts, or honey [58, 66].

The mixtures impregnating the mummy bandages of the older child (KMM A 64) differed slightly from those used to impregnate the bandages of the younger one (KMM A 63). In the case of the former, according to the ATR-FT-IR analysis, the use of plant oil and maybe the addition of wax on the wrapping and stuffing textiles, in some instances addition of resin, and traces of long chain hydrocarbon mineral oil were noted, together with traces of salts, such as CaCO_3_, silicates, and sulphates. The results of ATR-FT-IR analysis of textiles from the younger mummy indicate that some resin and long C-H chain containing hydrocarbons, like mineral oil, were probably used. GC-MS analysis of solvent-extracted samples of the textile (sample YM S15), yielded aromatic compounds and resinous inclusions (Pinaceae resin?), clear signs of polysaccharides of pyranone, furanone (including arabinofuranose) and their derivatives. The latter could be related to natural plant gum or gum resin [60]. Also, it could be suggested that the likely origin of the main component is plant oil as due to the relatively higher abundance of palmitic acid (C_16:0_) over stearic acid (C_18:0_) [55], while the cholesterol could derive from the body. However, it is worth keeping in mind that, as the textile was directly attached to the body, it is somewhat difficult to distinguish between the embalming substances on the body, as opposed to those impregnating the textile, if indeed they were separately treated.

**Fig 7:**
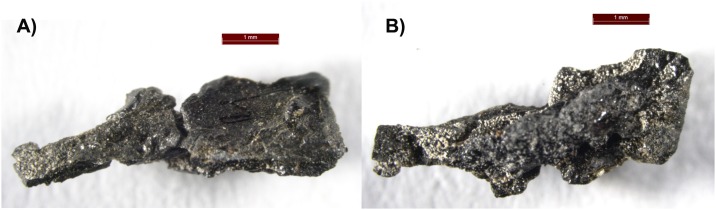
Optical microscope photos of the sample OM S7 from the left foot toe area showing the white particles (sodium salts) under the embalming resin sample. A—upper part, and B—lower part of the sample piece.

Finally, traces of salts were detected in the samples taken from the abdominal cavity and left foot of the older mummy. These small, white particles visible under the microscope (Fig 7) were noticed under the black embalming material and were initially located on the skin of the mummy. ATR-FT-IR and SEM-EDS analysis showed that they contain sodium sulphate (Na_2_SO_4_), some Fe-containing compound (maybe Fe-containing silicates) and traces of calcium carbonate. SEM-EDS analysis of a sample from the abdominal cavity (OM S4) showed that Na_2_SO_4_, probably sodium chlorite (NaClO_2_) and very small traces of mineral impurities, were present. Sodium sulphate and other sodium salts like sodium carbonate, sodium bicarbonate and sodium chloride are in the composition of a naturally occurring salt called natron, which was used as a dehydrating agent for drying the body during mummification [59, 67]. Inorganic substances were also detected on and under the nail of the younger mummy with the EDS spectra indicating the iron-containing silicates (elements Fe, Al, Si, O), different sulphates (e.g. BaSO_4_) and carbonates (CaCO_3_). Taken together, these might be indicative of possible salt-based body handling as part of the mummification process. Additionally, some phosphates and chlorine-containing compounds were also detected.

### Textiles

A total of 876 detached textile fragments (roughly 8.6 m^2^) and bundles of yarn stored together with the mummies were obviously the remains of their wrappings. Most of the bandages had been removed from the bodies already during earlier studies, regrettably without any documentation. In a few places cloth fragments still attached onto the mummies via embalming materials could be seen. The present amount of material indicates that the collection of textiles is incomplete, as there is insufficient fabric to wrap two bodies [68]. It is impossible to attribute these fragments to one or the other child and it cannot be excluded that some of these fabrics belonged to the animal mummies from the same collection. Almost all textiles are fragments from strips/bands suitable for wrapping, with the longest preserved piece measuring 4252 × 50 mm. In 63 cases tailoring elements, such as seams and stitching, sometimes joining different fabrics, proved that most of the textiles were reused for mummification. Recycling old cloth and clothing for mummy bandaging was a common practice [12, 68, 69], although new linen woven especially for specific funerary rites became more popular in the Graeco-Roman Period [39]. However, the majority of those pieces of linen tended to be shrouds.

**Fig 8:**
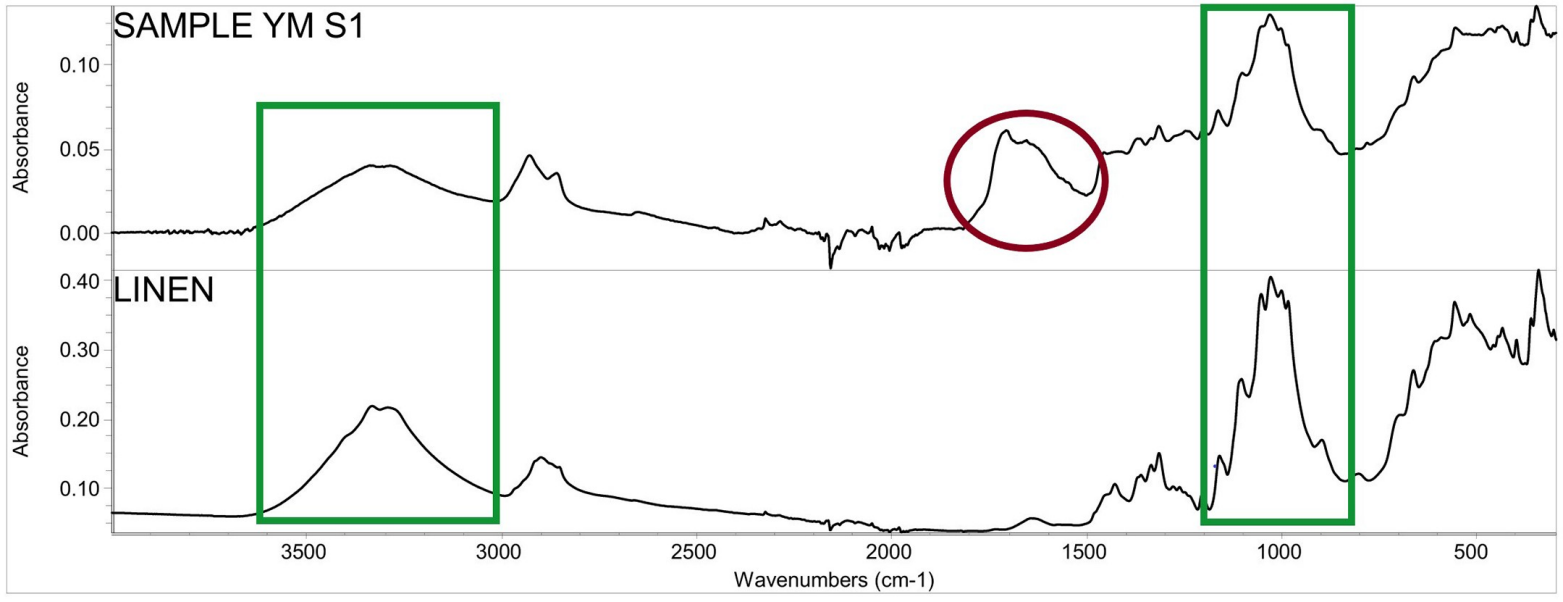
ATR-FT-IR spectra of textile samples from mummy (YM S1) and pure linen for comparison. Green boxes show the characteristic absorbance bands of cellulose-based fibres and the red box shows the area of spectrum that does not belong to fibres (could belong to embalming materials).

**Fig 9:**
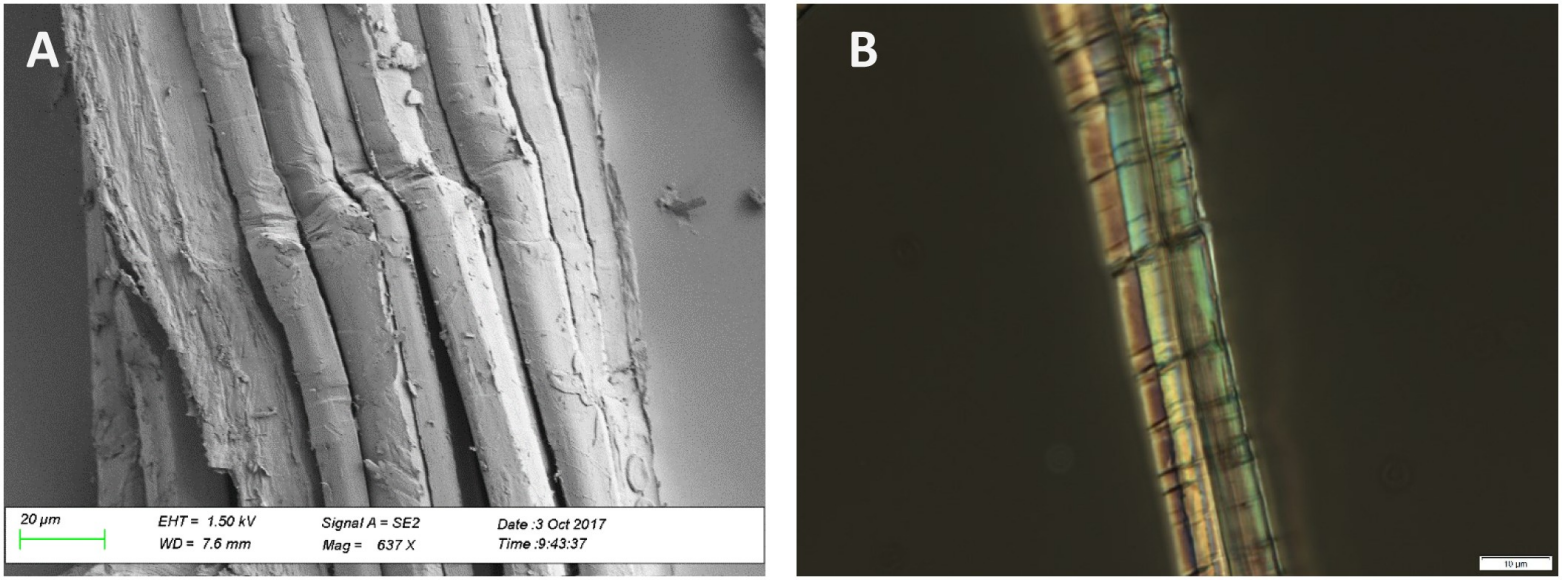
Dislocations in a line reveal that flax fibres were not entirely separated from each other during the preparation process. A—OM S5; SEM photo taken in Aalto University Nanomicroscopy Center; B—YM S12; TLM photo taken in the University of Tartu, Department of Archaeology.

Thirteen samples were also collected for fibre studies (Table 5). Those textiles attached to the mummies with a clear context were preferred. Yarn pieces up to approximately 8 mm in length were cut from the chosen textiles. From all of the ATR-FT-IR spectra (e.g. in Fig 8), characteristic absorption bands of cellulose-based fibres were found. Microscopic studies visualised well-preserved bast fibres that had characteristic nodes and dislocations, and oval to hexagonal cross sections, typical for flax fibres [30, 31] which were the usual textile used in Egypt. The modified Herzog test proved that the internal orientation of the fibres follows an S-direction, which provides an additional proof for identification as flax [29]. The result is not surprising as linen was employed almost exclusively for wrapping mummies [39] as well as for most clothing. The fibres are often still in the bundles having dislocations and nodes in a line (Fig 9). Thus, the fibres were not thoroughly processed (e.g. heavy retting, hackling, and combing known from historical periods) to achieve complete separation before spinning [70].

**Table 5:** List of samples and analyses conducted on textile fragments^a^.

Sample ID	Context and description	Analyses
OM S3	Tabby from abdominal cavity	TLM, PLM, ATR-FT-IR + PCA
OM S5	Yarns from abdominal cavity	TLM, PLM, SEM
OM S19	Tabby below the body	TLM, PLM
YM S1	Tabby stuck on the base of the skull	TLM, PLM, ATR-FT-IR + PCA
YM S3	Coarse tabby on the left leg	TLM, PLM, ATR-FT-IR + PCA
YM S4	Fine tabby on the left leg	TLM, PLM, SEM, ATR-FT-IR + PCA
YMS6	Tabby stuck on the base of the skull	TLM, PLM
YM S8	Tabby on the left side of the skull	TLM, PLM
YM S12	Tabby from the abdominal cavity	TLM, PLM
T S1	Tabby with drawing	TLM, PLM, ATR-FT-IR + PCA
T S2	Tabbies with seams	TLM, PLM
T S3	Basket weave	TLM, PLM
T S4	Tabby	TLM, PLM

^a^ TLM—transmitted light microscopy, PLM—polarised light microscopy, SEM—scanning electron microscopy, ATR-FT-IR + PCA—ATR-FT-IR spectroscopy and principal component analysis.

Except for nine items of basket weave with paired threads in both systems, all of the fragments were undyed tabbies. These represent invariably faced tabby weaves, which means that one yarn system is more densely placed than the other. The thread count varied, ranging from nine to 40 threads per one centimetre in the first yarn system and to 20 in the other (on average 24 and 10 respectively, a thread ratio of 2:1 or 2:3 being very common), indicating different qualities of cloth used. Nine fragments were fringed and four fragments had looped edges. Groups of self-bands or texture strips made of multiple or thicker threads inserted into the cloth occurred frequently. This mixture of cloth qualities is quite commonly found on Egyptian mummies [71].

All the threads in the fabrics were single and spun in an S-direction, and the sewing threads were usually plied (Z2s). Frequent knots, for the purpose of joining thread ends due to breakage or yarn shortage, were observed while studying the yarns in the detached textiles. Occasionally, spliced continuations occurred. Nevertheless, this does not indicate the splice-and-twist-technique used for yarn production in pharaonic Egypt [72], as this technique generally ceased by the second half of the first millennium BC [70]. The single and even threads with an angle of twist over 10° and lack of epidermis remaining in microscopy samples indicated that draft spinning was used for making yarns [70]. In conclusion, the results of the technical analysis [73] are in accordance with our knowledge about Egyptian textile production of the period [71, 74, 75].

### Insect remains

**Fig 10:**
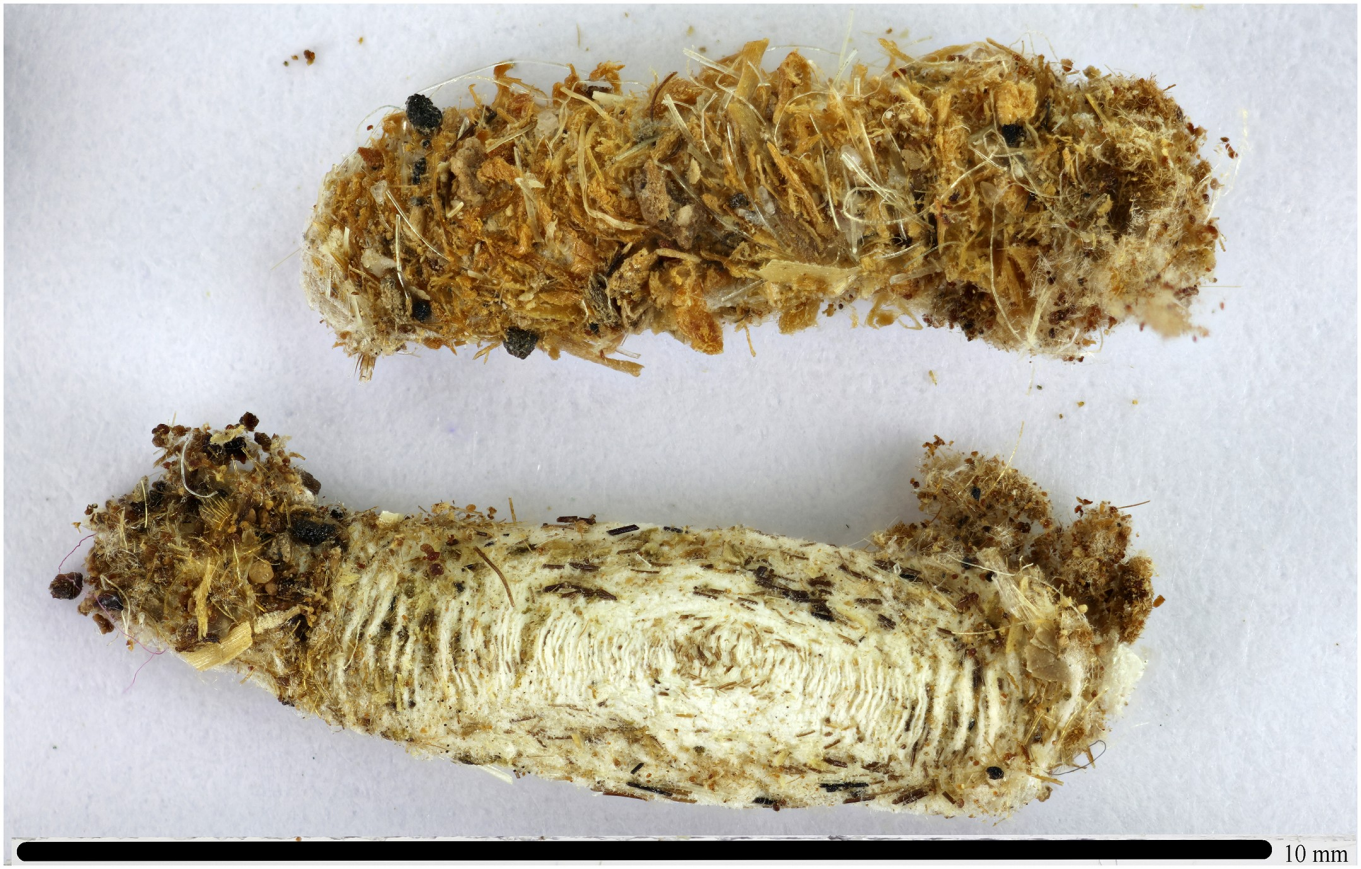
Cocoon remains recovered from the older mummy (KMM A 64) coffin.

Insect remains were collected under, and in between, the textile fragments located at the bottom of the older mummy’s (KMM A 64) exhibition coffin. Two cocoons (Fig 10) probably belong to the family Tineidae (order Lepidoptera), possibly representing two different species. The first whitish flattened cocoon (8.2 mm long) is tentatively identified as *Tinea pellionella* L. The second cocoon, reddish to light brownish (7.5 mm long), remains unidentified, but is very likely to also be from the Tineidae. The first cocoon was consistent with the external morphology of cases of *T*. *pellionella* (Case-bearing Clothes Moth), but was a bit smaller than its typical size range, i.e. 10–15 mm. Its outside surface was mostly matte and whitish and exhibited a sharp keel on either side, and a silk spinning pattern of near concentric ellipses followed the outline of each flattened side (with the pattern repeated on the inside surface of the cocoon), just as in *T*. *pellionella*. A range of particles (sand) and fibres were attached at either end together with a probable piece of insect cuticle apparently bearing long scales, and a range of other vegetable matter (including a possible fragment of papyrus). It is possible but not verified that mummy wrapping fabrics were included in the first cocoon. Inside this cocoon was a likely final instar head capsule attached to larval skin, with at least the sclerotized pronotum also visible. The maximum diameter of this slightly distorted head capsule was 0.88–1.08 mm, so as the sclerites would have been aligned more closely together in the living caterpillar, the actual size should therefore fit within the 0.60–0.88 mm range given (without sample size) for the final instar of the larva of modern examples of *T*. *pellionella* by Name and Bumroongsook [76].

The smaller cocoon had a rather untidy mixture of dark particles, transparent fibres/hairs and brown chips on its surface and the silk on the inside lacked the pattern of elliptical laying down of silk threads shown by the first cocoon. It contained two unidentified small objects: possibly a chip of clay, and a darkened sclerotized and rounded structure, possibly insect remains, not certainly derived from Lepidoptera.

The beetle fragments belong to a single species of the genus *Attagenus* Latreille, 1802 (Dermestidae). These are skin/carpet beetles feeding as larvae on dry animal matter such as, in the case of *Attagenus*, wool/cloth/hair/feathers etc., though larvae will eat meat and dead insects as well. Several of those species are known in Egyptian mummies [77]. As no fragments of adults or pupae, or dead larvae, are present, species level identification is unfortunately impossible.

There is little published information on Lepidoptera specimens found in Egyptian mummies, as most of the available data concerns Coleoptera [77, 78]. Thus, identification of one cocoon representing a species of *Tinea* (Tineidae), most likely *T*. *pellionella* if not a very closely related species, is noteworthy. A remaining question is the date and relation of the cocoons and beetle fragments with the mummy, i.e. are they contemporary with the body or do they represent a later infestation by the moths and beetles? In the case of cocoons, some fibres resemble in colour and size the ones found in the mummy, but there are a number of objects possibly of other origins. It should be noted that tineid larvae feed on keratin or keratin-containing materials [79], including wool, human and animal hair, and other insect fragments, and not on plant material. Thus, they may either represent a subsequent addition to the recently embalmed mummies, or a much later addition from the curational context still feeding on ancient material. The same applies to beetles, since similar species of *Attagenus* occur in both Egypt and Estonia, and may enter tombs or museums to feed on a suitable substrate. Therefore, it is presently impossible to distinguish whether these remains are from the initial deposition, or later additions related to museum storage and exhibition conditions.

## Conclusion

This study has furthered our understanding of the two child mummies in the museum collection. Firstly, the chronology, embalming technique and bandages, together with genetic data, all suggest an Egyptian provenance, confirming the available historical sources. From a palaeopathological viewpoint, we were only able to note nonspecific stress markers on the older mummy (KM A 64), namely the enamel hypoplasia and Harris lines, indicative of poor diet or disease, as well as a possible cyst on its mandible, leaving the precise cause of death still unknown [37, 38]. As far as mummification is concerned, it is clear that excerebration was achieved trans-nasally, a feature of Egyptian embalming since the New Kingdom [80]. This is especially important to note, because in the Graeco-Roman Period this practice could be waived, especially among children [39]. However, evisceration was still common, as is clearly evidenced by the flank incisions present on both mummies. It is quite possible that the viscera were returned to the body in wrapped packages, although this was more common in the 21st to 25th Dynasties [12]. However, the packages in the torsos of the two mummies suggest that this tradition might have continued. The absence of the heart in both children is curious as traditionally it was left in the body as it played a key role in the attainment of an Afterlife, both for adults and children. It is possible that the hearts of both these mummies are so covered with resin that they could not be identified, or it is conceivable that the hearts were removed inadvertently and never returned to the body, as has been found in other Egyptian mummies [81].

The initial body positions of the subjects varied as seen in other samples of the period [12]. These indicate differences in the wrapping techniques and in the ways these children had been handled before and after the dehydration of soft tissues. In addition, the male sex of both subjects appears to be in accordance with other data, which shows that the practice of preserving dead non-adults appears to favour males [42].

The embalming materials that were used for these mummies are in keeping with other mummies of this time period [59, 82]. Degraded plant oils and/or animal fats were detected as the main components with clear inclusion of resinous compounds, most likely relating to Pinaceae resins and maybe some pitches, various aromatic compounds, and, interestingly, polysaccharides (plant gum?). Furthermore, inclusion of waxes (most likely beeswax) was also detected. As additional and previously less detected constituents, traces of ricinoleic acid (castor oil), coumarin (galbanum) and probably bituminous material, were present as well. The latter is of interest as previously little evidence for this material has been identified in embalming materials, although Greek texts refer to it as a significant component of mummification [39]. It is noteworthy that the analytical results from samples of the head and body area, as well as textile-impregnating materials revealed slight differences in their components—perhaps specific solutions were used for different parts of the body as part of the mummification ritual. Also, we were able to identify traces of various salts most probably used for desiccating the body [54].

The textiles are also consistent with Egyptian mummies. The inscribed bandages (see above) and the use of linen amulets [14] support the AMS dating, and serve to narrow it a bit further, placing the mummies in the Ptolemaic era.

The previous destructive activities carried out on these bodies do not allow us to reconstruct precisely the pattern, if any, of stuffing and wrapping the body, but we could identify that at least partly reused linen was used for wrapping. Those in the body of the younger mummy may have served to give it more shape, and quite possibly the four packages in the other body correspond to the four different viscera that were removed traditionally: lungs, liver, intestines and stomach.

Thus, our study has provided an extensive body of new information on the two child mummies, by both reconstructing their biological profiles and investigating their provenance and mummification of these items, adding not only to the data available on Egyptian mummies now kept in the Baltic states [83–85], but also to the corpus of child mummies from ancient Egypt.

## Supporting information

S1 Appendix A Supplementary material on ancient DNA analysis. (DOCX)

S2 Appendix B Supplementary material on chemical residue analysis. (DOCX)

S3 Appendix C Supplementary material on 3D modelling. (DOCX)
