# Supplementary material for: Multidisciplinary investigation of two Egyptian child mummies curated at the University of Tartu Art Museum, Estonia (Late/Graeco-Roman Periods)
Source: PLoS One. 2020 Jan 16;15(1):e0227446. doi: 10.1371/journal.pone.0227446 (PMC6964855; doi:10.1371/journal.pone.0227446)
Supplement: S1 Appendix A — (DOCX) [file pone.0227446.s001.docx]

**S1. Appendix A. Supplementary Material on aDNA analysis**

The tooth was cut in half and only the root was used, the samples were briefly soaked in a 6% (tooth root) or 0.5% (hair) bleach solution, then rinsed with water and ethanol and left to dry under a UV light for an hour. The samples were left to digest in a buffer containing only 2.5 mM EDTA (pH 8.0) and proteinase K (tooth root) or also 10 mM Tris buffer (pH 8.0), 10 mM NaCl, 5 mM CaCl_2_, 2% SDS and 1 M DTT (hair) for 3 (tooth root) or 1 (hair) night(s) at 20˚C (tooth root) or 55˚C (hair). The digestion mixtures were centrifuged for 5 minutes at full speed to pellet undigested material. The supernatants were concentrated to a volume of 250 μl (Amicon Ultra-15 Centrifugal Filter Concentrator 15 ml 30K, Millipore) and the mixtures were purified (columns from High Pure Viral Nucleic Acid Large Volume Kit, Roche; buffers from MinElute PCR Purification Kit, QIAGEN). Next-generation sequencing libraries were built using NEBNext DNA Library Prep Master Mix Set for 454 (E6070, New England Biolabs) and Illumina-specific adaptors[1] following established protocols[1]. The universal sequencing primer and indexed primers were added by PCR using HGS Diamond Taq polymerase (Eurogentec). The mixtures were purified after end repair, adapter ligation and PCR (MinElute PCR Purification Kit, QIAGEN). The success of the extraction and library preparation was verified with Qubit (ThermoFisher), Fragment Analyser (Agilent Technologies) and qPCR. The libraries were sequenced at the UT, Institute of Genomics, Estonian Biocentre core lab with Illumina NextSeq 500 using a 75 bp single-end kit.

Before mapping, the sequences of adaptors and indexes were cut from the ends of DNA sequences using cutadapt 1.11[2]. Sequences shorter than 30 bp were also removed to avoid random mapping of sequences from other species. The sequences were mapped to human reference sequence GRCh37 using Burrows-Wheeler Aligner (BWA 0.7.12)[3] and command mem. After mapping, the sequences were converted to BAM format and sequences that did not map to the reference sequence were removed with samtools 1.3[4]. Indels were realigned and the base quality score was recalibrated using Genome Analysis Toolkit (GATK 3.5)[5]. Then the lanes were merged and PCR duplicates were removed with Picard MarkDuplicates (<http://broadinstitute.github.io/picard/)> and indels were realigned again with GATK[5]. Finally, reads with mapping quality below 10 were filtered out. Mitochondrial DNA haplogroups were determined using mtDNA-Server (<https://mtdna-server.uibk.ac.at/index.html)> and checked manually.

As a result of degrading over time aDNA can be distinguished from modern DNA by certain characteristics: short fragments with long single-stranded overhangs and a high frequency of C=>T substitutions at the 5' ends of sequences due to cytosine deamination. The program mapDamage2.0 [6] was used to estimate the frequency of 5' C=>T transitions.

**References**

1. Meyer M, Kircher M. Illumina sequencing library preparation for highly multiplexed target capture and sequencing. Cold Spring Harb Protoc. 2010; 2010: 5448.

2. Martin M. Cutadapt removes adapter sequences from high-throughput sequencing reads. EMBnet.journal. 2011; 17: 10–12.

3. Li H, Durbin R. Fast and accurate short read alignment with Burrows-Wheeler transform. Bioinforma Oxf Engl. 2009; 25: 1754–1760.

4. Li H, Handsaker B, Wysoker A, Fennell T, Ruan J, Homer N, et al. The Sequence Alignment/Map format and SAMtools. Bioinforma Oxf Engl. 2009; 25: 2078–2079.

5. McKenna A, Hanna M, Banks E, Sivachenko A, Cibulskis K, Kernytsky A, et al. The Genome Analysis Toolkit: a MapReduce framework for analyzing next-generation DNA sequencing data. Genome Res. 2010; 20: 1297–1303.

6. Jónsson H, Ginolhac A, Schubert M, Johnson PLF, Orlando L. mapDamage2.0: fast approximate Bayesian estimates of ancient DNA damage parameters. Bioinformatics. 2013; 29: 1682–1684.
