# Supplementary material for: Multidisciplinary investigation of two Egyptian child mummies curated at the University of Tartu Art Museum, Estonia (Late/Graeco-Roman Periods)
Source: PLoS One. 2020 Jan 16;15(1):e0227446. doi: 10.1371/journal.pone.0227446 (PMC6964855; doi:10.1371/journal.pone.0227446)
Supplement: S2 Appendix B — (DOCX) [file pone.0227446.s002.docx]

**S2. Appendix B. Supplementary Material on chemical residue analysis**

**Optical Stereomicroscopy**

For the analysis of the samples Leica M165 FC stereomicroscope was used with range of magnification of 7.3–120. It is possible to use instrument in under and side lighting regime. Microscope was connected to computer and Leica Application Suite (LAS) version 3.7.0 was used for image processing.

**ATR-FT-IR spectroscopy**

All ATR-FT-IR spectra were recorded using a Thermo Scientific Nicolet 6700 FT-IR spectrometer equipped with a Smart Orbit diamond micro-ATR accessory. The spectrometer has DLaTGS detector, Vectra Aluminium Interferometer, a sealed and desiccated optical bench with CsI optics. The FT-IR spectrometer is protected from atmospheric moisture by constant purge with dry air.

Mostly solid samples did not need any special preparation, these were used as received. However, impregnating material from the textiles were extracted with DCM solvent.

The solid sample was placed directly on the ATR crystal, was pressed against the ATR crystal and the ATR-FT-IR spectrum was scanned. In case of solutions, a drop of solution was placed on the ATR-crystal, solvent was evaporated and IR spectrum from the extract was recorded.

During the measurement resolution was 4 cm^-1^, wavenumber range was 4000-225 cm^-1^, number of scans was 256, zero filling factor was 0 and apodization window was Happ-Genzel. Thermo Electron’s OMNIC 9 software was used to collect and process the IR spectra.

**GC-MS**

Sample was prepared following solvent extraction method. Sample (direct residue of embalming material or textile fragments, see Table S2.1 for details) was suspended in 2 ml of dichloromethane (DCM) and methanol (2:1 v/v) mixture, ultrasonificated for 15 min, centrifuged and solvent soluble fraction removed with Pasteur pipette. The same procedure was repeated three times to obtain the total lipid extract (TLE). The solvent was evaporated under a gentle stream of nitrogen. Silyl derivatives were created adding 50 µl of N,O-bis(trimethylsilyl)trifluoroacetamide (BSTFA) with 1% TMCS into the TLE, followed by heating (70 °C, 1 hour). The excess BSTFA was removed under a gentle stream of nitrogen and sample was rediluted with DCM.

GC-MS analysis was conducted with Agilent 7890A Series gas chromatography and Agilent 5975C Inert XL mass-selective detector with a DB5-MS (5%-phenyl)-methylpolysiloxane column (30 m × 0.25 mm × 0.25 µm). Injected sample size was 1 µl. The splitless injector and interface were maintained at 300 °C and 280 °C respectively, helium was used as the carrier gas at a constant flow. The GC column was inserted directly into the ion source of the mass spectrometer. The ionization energy was 70 eV and spectra were obtained by scanning between m/z 50 and 800 amu. The temperature program was set as follows: 50 °C for 2 min, thereafter gradient of 10 °C/ min up to 325 °C for 6.5 min. Compounds were identified with Agilent Chemstation software using also NIST mass spectra library.

**ESI-FT-ICR-MS**

Small amount of sample (app. 2 mg) was dissolved in 0.5 ml of a mixture of methanol and dichloromethane (1:1) that additionally contained a small amount (around 0.1 μM) of phosphazenes as internal calibration standards. The positive ions of the following compounds were used for internal calibration: H-P_1_(pyrr)_3_·HBF_4_ (*m/z* 257.18896), 4-CF_3_-PhP_1_(pyrr) (*m/z* 401.207643), 2-Cl-C_6_H_4_-P_2_(pyrr)_5_·HBPh_4_ (*m/z* 552.28947), Ph(CH_3_)CH-P_4_(dma)_9_·HBF_4_ (*m/z* 682.43526), Ph(CH_3_)CH-P_4_(pyrr)_9_·HBF_4_ (*m/z* 916.57611). (Teearu et. al 2017)

The solution was sonicated in the heated water bath at 35 °C for 15-20 minutes. The sample solution was filtered through a Whatman^TM^ syringe filter (PTFE, 13 mm, pore size 0.45 μm) and analysed.

The high resolution mass spectra were obtained on Varian 910-FT-ICR-MS that has a 7 Tesla superconducting magnet and can be coupled with several ionisation sources, incl. electrospray ionisation source (ESI). The flow rate used for the infusing of the sample solutions was 10 μml/min. The following ionization parameters were used: spray chamber temperature 30 °C, nebulizing gas (N_2_) pressure 50 psi at 350 °C, shield voltage 400 V. The *m/z* range under observation was *m/z* 200 – *m/z* 950.

**SEM-EDS**

Analysis were performed using variable pressure Zeiss EVO MA15 scanning electron microscope (SEM), equipped with Oxford X-MAX energy dispersive detector system (EDS). Backscattered electron detector (BSD) was used. For the analyses, samples were placed on the sticky carbon conductive tab. Samples were uncoated and studied in variable pressure mode using 20 keV accelerating voltage and focused electron beam. Spectra were afterwards processed using Aztec software.

**Table S2.1. GC-MS identified compounds.^a^**

| **Sample ID** | **Material** | **Sample area** | **General description** |
| --- | --- | --- | --- |
| **Older mummy (KMM A 64)** | | | |
| OM S4 | Embalming resin | Abdominal cavity | SFA (C_5:0-24:0_), DC (C_4-12, C14, 16, 18_), OFA (C_5-8, 10_), UFA (C_18:2_), phenolic acids (benzoic acid), ALK(C_27_, C_29_, C_44_), phy, chol, abie |
| OM S10 | Embalming resin | On the right ear | SFA (C_5:0-28:0_), DC (C_5-12_), OFA (C_18_), UFA (C_18:1, 18:2_), ALK(C_27_), HFA (C_18_), phenolic acids (benzoic acid, cinnamic acid), polysaccharides (pyranone, furanone & derivates), coumarin |
| **Younger mummy (KMM A 63)** | | | |
| YM S14 | Embalming resin | On the left temple | SFA (C_6:0-22:0_), DC (C_5-12, 14_), OFA (C_8, 10, 16_), UFA (C_16:1, 18:1, 18:2_), phenolic acids (benzoic acid), abie, phen, chol, ricinoleic acid |
| YM S15 | Soaked textile (bandage) fragment | Under the base of left foot | SFA (C_5:0-20:0_), DC (C_2, 5-11_), OFA (C_3, 6-7_), UFA (C_4:1, 14:1, 16:1, 18:1, 18:2_), phenolic acids (benzoic acid), polysaccharides (pyranone, furanone (arabinofuranose) & derivates), abie, phen, chol |

^a^(Cn:x) - carboxilic acids with carbon length n and number of unsaturations x, SFA – saturated fatty acid, UFA – unsaturated fatty acids, DC - α,ω-dicarboxylic acids, OFA –oxy fatty acids, HFA – hydroxy fatty acids, ALK – alkanes, chol – cholesterol or derivative, phy – phytanic acid, phen – 1-phenanthrene carboxylic acid.
