# Supplementary material for: Multidisciplinary investigation of two Egyptian child mummies curated at the University of Tartu Art Museum, Estonia (Late/Graeco-Roman Periods)
Source: PLoS One. 2020 Jan 16;15(1):e0227446. doi: 10.1371/journal.pone.0227446 (PMC6964855; doi:10.1371/journal.pone.0227446)
Supplement: S3 Appendix C — (DOCX) [file pone.0227446.s003.docx]

**S3. Appendix C. Supplementary Material on 3D modelling**

The model for the younger mummy was made out of 167 aligned photos, which were all masked to remove the background. The only complication of making the model was the blind spot in the neck and knee area, where the mummy was supported (See 3D model of a younger mummy at link below). In hindsight, while photographing the back two sets of pictures should have been made with different locations of the plexiglass support.

The modelling of the older mummy proved to be more difficult. There were many unfocused areas in the pictures, especially around the feet, which meant that these areas had to be masked as well. Only 125 photos out of 151 were aligned for the model of the mummy lying on its stomach. The 177 photos out of 182 aligned for the model when lying on its back. However, when the two models were separately aligned, the programme had no difficulties in merging them into one model. The final model had some blind areas, under its crossed arms and between the feet, but otherwise the result was good.

The main lesson with modelling is that the best practice is to combine the two methods. A supported mummy can be photographed from almost every angle. It could then be flipped and the blind supported areas would also be captured and later added to the model. Also, for areas such as legs that need a higher depth of field, the lowest aperture setting combined with a tripod should be used.

**3D Models:**

Older mummy (KMM A 64): <https://skfb.ly/6u8Hu>

Younger mummy (KMM A 63): <https://skfb.ly/6HrZO>
